# The Current Role of Decompressive Shunts and Liver Transplant in Portal Hypertension

**DOI:** 10.1155/1991/79478

**Published:** 1991

**Authors:** J. Michael Henderson

**Affiliations:** Department of Surgery Emory University School of Medicine Atlanta Georgia 30322 USA


*HPB Surgery*, 1991. Vol. 4, pp 27-32
Reprints available directly from the publisher
Photocopying permitted by license only

© 1991 Harwood Academic Publishers GmbH
Printed in the United Kingdom

### THE CURRENT ROLE OF DECOMPRESSIVE SHUNTS AND LIVER TRANSPLANT IN PORTAL HYPERTENSION

J. MICHAEL HENDERSON
*Department of Surgery, Emory University School of Medicine, Atlanta, Georgia 30322*

*(Received 17 September 1990)*

KEY WORDS: Portosystemic shunts, liver transplant, distal splenorenal shunt

### INTRODUCTION

Surgical rescue has become a catch phrase for the Surgeon's role in management of variceal bleeding[1]. This is largely because of the current popularity of pharmacologic portal pressure reduction and the widespread use of endoscopic sclerotherapy. These two therapies both reduce the risk of rebleeding from varices by approximately 50%, but even then 30–40% of patients will rebleed through propranolol and/or sclerotherapy. Surgical options are reserved for patients who rebleed through these therapies or alternatively for patients in whom the high rebleeding risk is unacceptable.

This paper addresses the current role of decompressive shunts and liver transplantation in management of variceal bleeding.

### *Patient Evaluation*

Acute variceal bleeding can usually be stopped by endoscopic sclerotherapy. When stabilized the patient should be evaluated for the risk of further bleeding and the underlying cause of the portal hypertension. Emphasis of the latter should be on the severity of the underlying liver disease. Most deaths in patients with variceal bleeding are from liver failure.

History, physical examination, and standard laboratory tests of biochemical and haematologic indices provide the basis of evaluation. Ascites, encephalopathy and poor nutritional status indicate advanced disease, and when supported by elevated bilirubin, coagulopathy and hypoalbuminaemia suggest to a surgeon that the patient is a poor candidate for any operation other than a liver transplant. Endoscopy, vascular imaging techniques, liver biopsy and quantitative liver function testing can all be useful in evaluating the patient with more subtle disease, and in making therapy choices.

---

Correspondence to: J. Michael Henderson, FRCS, Department of Surgery, Emory University School of Medicine, Atlanta, Georgia 30322

*Decompressive Shunts*

These fall into three groups: total portal systemic shunts, partial portal decompression, and selective variceal decompression.

*Total shunts* are either the classic end-to-side portacaval shunt or a variety of side-to-side shunts such as portacaval, mesocaval, central splenorenal[2]. The latter decompress the hepatic sinusoids in addition to relieving splanchnic hypertension. All control variceal bleeding, but loss of portal perfusion accelerates liver failure.

Current data suggest that patients with alcoholic cirrhosis tolerate total shunts better than nonalcoholic paitents[3]. Continued massive variceal bleeding can be stopped by a total shunt: if subsequent transplant is a consideration a mesocaval shunt is preferred as it does not disturb the liver hilus. A further indication for a total portal systemic shunt is acute Budd-Chiari Syndrome when a side-to-side shunt decompresses the obstructed sinusoids and can stop the progression of hepatocyte necrosis[4].

*Partial Portal Decompression* can be achieved with an 8mm interposition portacaval H-graft[5]. At this diameter of shunt portal pressure is reduced to approximately 12 mm Hg, which is similar to the level of portal pressure below which no bleeding occurs in the pharmacologic studies. Portal perfusion is maintained in 82% of patients with this diameter of shunt.

Currently, more data needs to be collected on these shunts, and controlled studies are required to confirm their haemodynamic efficacy and clinical role.

*Selective variceal decompression* is most commonly achieved by distal splenorenal shunt (DSRS)[6]. Control of variceal bleeding is as good with selective shunt as a total shunt, and is achieved in approximately 90% of patients[2]. The physiologic goal of maintaining portal perfusion is significantly better achieved in nonalcoholic (90%) patients than alcoholic (50%) after standard DSRS[7]. Addition of splenopancreatic disconnection, taking the entire splenic vein out of the pancreas, improves maintenance of portal flow in alcoholics to 84%[8]. Survival parallels these portal perfusion differences, being significantly better for non-alcoholics compared to alcoholics after standard DSRS[9,10], and being improved in alcoholic patients by splenopancreatic disconnection[8] (Figure 1.)

Prospective randomized clinical trials comparing DSRS to a variety of total shunts in predominantly (83%) alcoholic patients shows similar control of bleeding, operative mortality and longterm survival[2,3,11].

*Sclerotherapy Versus Shunt*

Five prospective randomized trials have compared endoscopic sclerotherapy to shunt surgery[12–16]. The common finding to these studies was improved control of variceal bleeding with shunt surgery. The data on survival is less clear.

The first study[12] compared sclerotherapy to emergency portacaval shunt in patients with alcoholic cirrhosis and acute variceal bleeding. Both early and late control of bleeding was better in the shunt group. Survival was not significantly different between groups, but a feature of this study was the role of surgical rescue of patients with persistent or late rebleeding from varices.

The other four studies compared sclerotherapy to distal splenorenal shunt. The consistent feature was significantly better bleeding control in the DSRS group. Three studies[14–16] showed no significant difference in survival in the two groups,

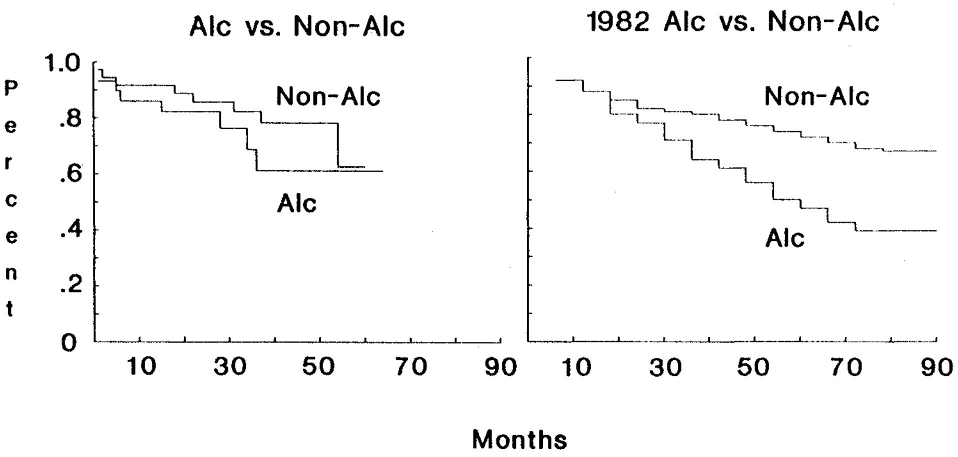

**Figure 1** Alcoholic versus nonalcoholic cirrhosis survival patterns after distal splenorenal shunt. On the right, alcoholic cirrhotic patients have significantly poorer survival than nonalcoholic patients after standard DSRS, while on the left this difference is not seen after DSRS with splenopancreatic disconnection. (Reproduced with permission from *Annals of Surgery*, 1989; 210:332–341.

while one showed improved survival in the group randomized to sclerotherapy[13]. This latter study[13], from our group, again emphasizes the importance of appropriate surgical rescue therapy in patients who fail sclerotherapy. Thirty five percent of patients failed sclerotherapy, and it was the successful surgical salvage of these patients who made the difference in survival between these groups. Further, this survival advantage was seen only in patients with alcoholic cirrhosis (Figure 2) supporting a treatment strategy of initial sclerotherapy with decompressive shunt reserved for those with rebleeding. In the nonalcoholic patients, the similar survival in the two groups combined with the significantly better bleeding control with DSRS, favors shunt as primary therapy.

### *Liver Transplantation*

The evolution of liver transplantation to a viable clinical therapy[17] in the past decade means that it must always be considered in management decisions. The indication for this therapy is end-stage liver disease. Variceal bleeding is certainly one of the more lethal complications of cirrhosis and portal hypertension, but not all patients with variceal bleeding have end-stage disease. Several papers or reviews have addressed the role of liver transplant in management of variceal bleeding[18–21].

Clearly, not all pateints with variceal bleeding can or should be managed by liver transplantation. While the concept of a curative rather than a palliative procedure for such patients is attractive, the risk: benefit ratio must be weighed for each patient. The decision to transplant a patient with variceal bleeding is easy if they are Child's Class C and otherwise fulfill transplant criteria. Equally, the decision to *not* transplant should be easy for good risk Child's A patients in whom lesser therapies effective in control of variceal bleeding can be offered, with a relatively low risk of rapid progression to liver failure. More problematic are the middle risk

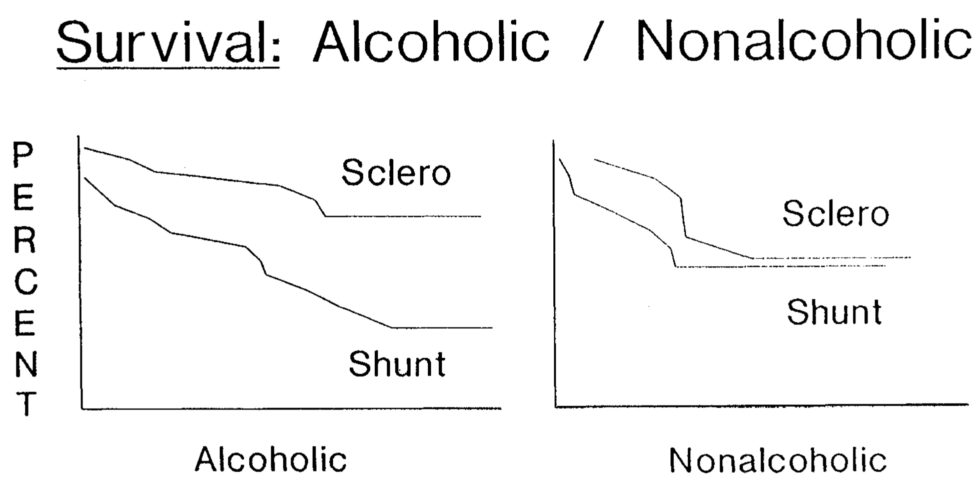

**Figure 2** The different survival pattern of alcoholic and nonalcoholic patients in the Emory prospective randomized trial[13] comparing DSRS and sclerotherapy. The group with significant survival advantage are the alcoholic cirrhosis patients who receive initial sclerotherapy with surgical rescue of the 35% who ultimately fail sclerosis.

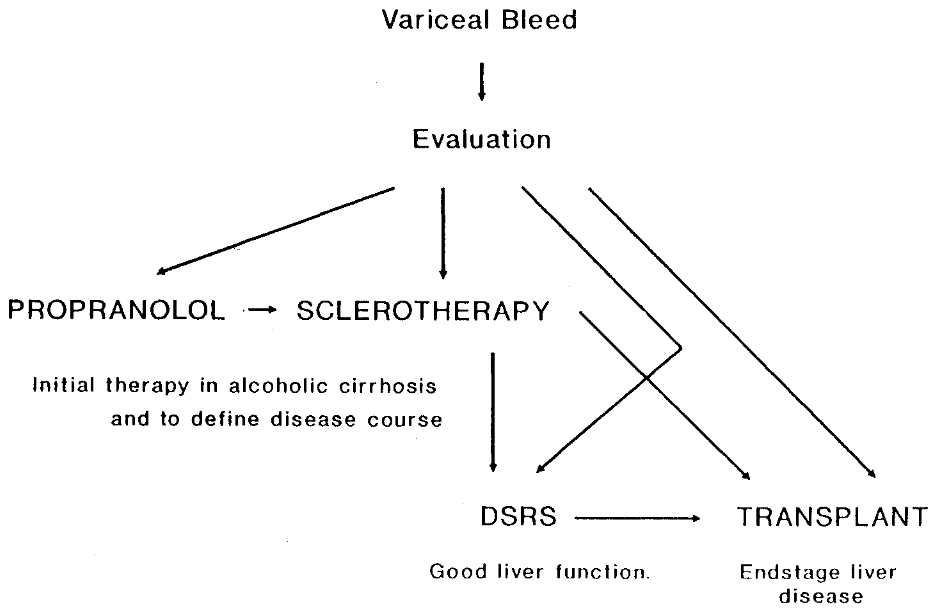

**Figure 3** Algorithm of management for prevention of recurrent variceal bleeding.

patients in whom the course of their liver disease may be less predictable: these patients should perhaps be the target or more objective hepatic function studies to facilitate making this decision.

What treatment should be used in patients in whom transplant is a likely ultimate step? Opinions vary. A lesser (and nonoperative) therapy will make subsequent transplant technically easier, but should a patient be offered transplant early because of variceal bleeding if their own liver is adequate when their bleeding has been controlled? If surgery is required urgently as a life saving measure, or indicated in an elective (and at this time, good risk) patient the two preferred choices are mesocaval shunt or DSRS.

### *Summary*

Based on our current management of many patients with variceal bleeding and the availability of all treatment options at our institution, the algorithm of management given in Figure 3 has evolved.

### *References*

1. Grace, N.D. (1990) Prevention of recurrent variceal bleeding: is surgical rescue the answer? *Annals of Internal Medicine*, **112**, 242–244
2. Langer, B., Taylor, B.R. and Greig P.D. (1990) Selective or total shunts for variceal bleeding. *American Journal of Surgery*, **160**, 75–79
3. Henderson, J.M. (1986) Variceal bleeding: which shunt? *Gastroenterology*, **91**, 1021–1023
4. Henderson, J.M. Warren, W.D., Millikan, W.J. *et al.* (1990) Surgical options, hematologic evaluation, and pathologic changes in Budd-Chiara Syndrome. *American Journal of Surgery*, **159**, 41–50
5. Sarfeh, I.J., Rypins, E.B. and Mason, G.R. (1986) A systematic appraisal of portacaval H-graft diameters. *Annals of Surgery*, **204**, 356–363
6. Warren, W.D., Zeppar, R. and Foman, J.S. (1967) Selective transplenic decompression of gastroesophageal varices by distal splenorenal shunt. *Annals of Surgery*, **166**, 437–448
7. Henderson, J.M., Gong-Liang, J., Galloway, J., Millikan, W.J. Jr., Sones, P.J. and Warren, W.D. (1985) Portaprival collaterals following distal splenorenal shunt: Incidence, magnitude and associated portal perfusion changes. *Journal of Hepatology*, **1**, 649–661
8. Henderson, J.M., Warren, W.D., Millikan, W.J., Galloway, J.R., Kawasaki and Kutner, M.H. (1989) Distal splenorenal shunt with splenopancreatic disconnection: a four year assessment. *Annals of Surgery*, **210**, 332–341
9. Zeppa, R., Hensley, G.T., Levi, J.U. *et al.* (1978) The comparative survival of alcoholics versus nonalcoholics after distal splenorenal shunt. *Annals of Surgery*, **187**, 510–514
10. Henderson, J.M., Millikan, W.J. Jr., Wright-Bacon, L., Kutner, M.H. and Warren, W.D. (1983) Hemodynamic differences between alcoholic and non-alcoholic cirrhotics following distal splenor-ental shunt – effect on survival? *Annals of Surgery*, **198**, 325–334
11. Rikkers, L.F. (1988) Is the distal splenorenal shunt better? *Hepatology*, **8**, 1705–1707
12. Cello, J.P., Grendell, J.H., Crass, R.A. *et al.* (1987) Endoscopic sclerotherapy versus portacaval shunt in patients with severe cirrhosis and acute variceal hemorrhage. Long-term follow-up. *New England Journal of Medicine*, **316**, 11–15
13. Henderson, J.M., Kutner, M.J., Millikan, W.J., Galambos, J.T., Brooks, W.S., Riepe, S.P., Bryan F.C. and Warren, W.D.(1990) Endoscopic variceal sclerosis compared with distal splenore-nal shunt to prevent recurrent variceal bleeding in cirrhosis. *Annals of Internal Medicine*, **112**, 262–269
14. Rikkers, L.F.,, Burnett, D.A., Volentine, G.D. *et al.* (1987) Shunt surgery versus endoscopic sclerotherapy for long-term treatment of variceal bleeding: early results of randomized trial. *Annals of Surgery*, **206**, 261–271
15. Teres, J., Bordas, J.M., Bravo, D. *et al.* (1987) Sclerotherpay vs distal splenorenal shunt in the elective treatment of variceal hemorrhage: a randomized controlled trial. *Hepatology*, **7**, 430–456

16. Spina, G.P, Santambrogio, R., Opocher, E. *et al.* (1990) Distal splenorenal shunt versus endoscopic sclerotherapy in the prevention of variceal bleeding. First stage of a randomized controlled trial. *Annals of Surgery*, **211**, 178–186
17. Starzl, T.E., Demetris, A.J., Van Thiel, D.H. (1989) Medical progress: Liver transplantation. *New England Journal of Medicine*, **321**, 1014–1022 and 1092–1099
18. Iwatsuki, S., Starzl, T.E., Todo, S. *et al.* (1988) Liver transplantation in the treatment of bleeding esophageal varices. *Surgery*, **104**, 697–705
19. Bismuth, H., Adam, R., Mathur, S. and Sherlock, D. (1990) Options for elective treatment of portal hypertension in cirrhotic patients in the transplantation era. *American Journal of Surgery*, **160**, 105–110
20. Wood, R.P., Shaw, B.W. Rikkers, L.F. (1990) Liver transplantation for variceal hemorrhage *Surgical Clinical of North America*, **70**, 449–461
21. Millikan, W.J., Henderson, J.M., Stewart, M.T., Warren, W.D. *et al.* (1989) Change in hepatic function, hemodynamics, and morphology after liver transplant: Physiological effect of therapy. *Annals of Surgery*, **209**, 513–525

